# Perspectives of Nanoparticles in Male Infertility: Evidence for Induced Abnormalities in Sperm Production

**DOI:** 10.3390/ijerph18041758

**Published:** 2021-02-11

**Authors:** Mehwish Iftikhar, Aasma Noureen, Muhammad Uzair, Farhat Jabeen, Mohamed Abdel Daim, Tiziana Cappello

**Affiliations:** 1 Department of Zoology, Government College University, Faisalabad 38000, Pakistan; mehwish135@yahoo.com (M.I.); farjabeen2004@yahoo.co.in (F.J.); 2 Department of Biology, Virtual University of Pakistan, Faisalabad 38000, Pakistan; 3 School of Life Sciences and Biotechnology, Shanghai Jiao Tong University, Shanghai 200240, China; uzairshakoor@gmail.com; 4 Department of Zoology, College of Science, King Saud University, Riyadh 11451, Saudi Arabia; 5 Department of Pharmacology, Faculty of Veterinary Medicine, Suez Canal University, Ismailia 41522, Egypt; abdeldaim.m@vet.suez.edu.eg; 6 Department of Chemical, Biological, Pharmaceutical and Environmental Sciences, University of Messina, 98166 Messina, Italy

**Keywords:** nanoparticles, nanotoxicology, reproductive health, spermatogenesis, sperm count, NP transfer

## Abstract

Advancement in the field of nanotechnology has prompted the need to elucidate the deleterious effects of nanoparticles (NPs) on reproductive health. Many studies have reported on the health safety issues related to NPs by investigating their exposure routes, deposition and toxic effects on different primary and secondary organs but few studies have focused on NPs’ deposition in reproductive organs. Noteworthy, even fewer studies have dealt with the toxic effects of NPs on reproductive indices and sperm parameters (such as sperm number, motility and morphology) by evaluating, for instance, the histopathology of seminiferous tubules and testosterone levels. To date, the research suggests that NPs can easily cross the blood testes barrier and, after accumulation in the testis, induce adverse effects on spermatogenesis. This review aims to summarize the available literature on the risks induced by NPs on the male reproductive system.

## 1. Introduction

Nanoparticles (NPs) are defined as particles having size less than 100 nm [1] and these particles can be 0D, 1D, 2D and 3D on the bases of their overall shape [2]. The importance of nano sized particles was enhanced when researchers found that size can alter the properties of a substance [1]. NPs are extensively used in industrial and biomedical sectors [3]. It is reported that there are more than 1814 products including textiles, antibiotics, sport and food items in which nano-sized particles are used and this number of products is rapidly increasing [3]. The immense growth in the advanced field of nanotechnologies with all its far reaching benefits has drawn the attention of researchers towards the health risks induced by NPs [4]. Throughout evolution, humans have been exposed to various airborne NPs but intensity of exposure is now significantly increased due to the diverse use of nanoparticles in products in our daily life [5]. This higher production rate of NPs also poses risks because of their release into the environment as nano structural materials that may exert their toxic impact on the ecosystem [6,7]. The hazard of NPs is directly or indirectly associated with the consumers that are exposed to these nanomaterials and their harmful effects during their usage [8]. In fact, NPs interact with the human body via ingestion through food, injection, penetration through skin and inhalation [9]. This uptake of NPs can be non-intentional (i.e., by inhalation, transdermal) and intentional (i.e., by injection, food additives, ingredients and supplements containing NPs) [10]. NPs then penetrate into body organs through the blood circulatory system [11] and interact with biological systems leading to intense cytotoxicity [12,13,14,15,16] due to their nano-size [17].

The ability of different chemicals to penetrate the cell is a matter of concern with regard to reproductive toxicity due to the complex biological processes that can be affected by these compounds through environmental exposure [18]. Reproductive toxicity is now considered as an important issue to be investigated in overall toxicology [10]. Fertility and successful reproduction are of vital importance to sustain a species and there is an increased need of public awareness because of NPs’ induced reproductive toxicity, since production of engineered nanoparticles might increase the risk of interference with the reproductive system [19]. The male reproductive system is considered sensitive to oxidative stress and inflammation [20,21,22], and both can be used as hallmarks for the exposure to NPs [20]. Oxidative stress is a major contributing factor to reproductive toxicity due to NPs [23]. Reactive oxygen species (ROS) is the key factor in inducing 30–80% of infertility issues in men [24], as the increased production of ROS leads to cell apoptosis and impaired spermatogenesis [25]. Several studies have reported NPs’ induced oxidative stress in male reproductive organs after the exposure to different nanoparticles such as Ag NPs [26,27], co-exposure of TiO_2_ NPs and ZnO NPs [28], Cu NPs [29] and Ni NPs [30]. Epidemiologists have taken a keen interest regarding these reproductive health issues because in some areas young males demonstrate a suboptimal quality and number of spermatozoa [31,32,33]. In recent years, sperm quality and numbers have been reduced in humans and in many cases the reason behind this is still unknown [34]. According to previous studies, NPs may affect spermatogenesis because of their presence in the environment, and those people who are mostly exposed to NPs are at major risk [34]. However, nanoparticles are not all involved in inducing adverse effects. In fact, Shi et al. [35] described that nano-selenium used as a supplement positively enhanced the quality of goat spermatozoa. Therefore, some nanoparticles showed nontoxic and beneficial effects on spermatogenesis. However, the route, dose, size and characteristics of NPs play vital roles in determining their impact on male germ cells [36]. NPs have the ability to cross the blood-testis barrier which increases the concern about biocompatibility and NP distribution [37].

The main object of this review is to provide a comprehensive summary of the available literature on nanoparticles’ deposition, translocation to the testes and induced reproductive toxicity, as well as to provide insights on the potential reproductive risks posed by various NPs on male germ line cells and spermatozoa.

## 2. Male Reproductive System and Potential

The male reproductive system comprises of primary (gonads/testes) and secondary (ducts/glands) and accessory, reproductive organs that assist in successful reproduction [38,39]. In order to assess male reproductive potential, the first step in a clinical evaluation is a detailed semen analysis. In regard to normal semen parameters, the World Health Organization (WHO) has revised the lower reference limits (95% confidence intervals) for the analysis of semen, which are listed in Table 1 [40].

**Figure 1:**
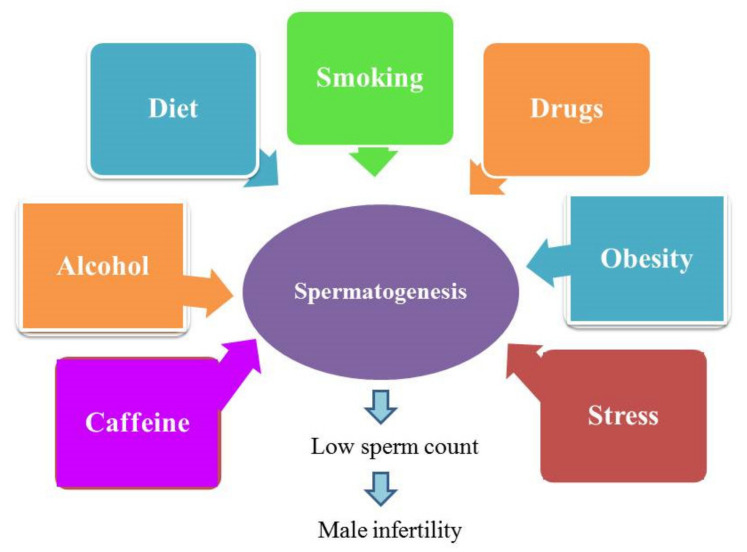
Lifestyle causes male infertility.

Among semen parameters, the assessment of sperm concentration is crucial in evaluating male reproductive potential for several reasons, including: (1) sperm count is importantly linked to male fertility and it is used as the first step in semen analysis towards the identification of factors behind male infertility [41,42]; (2) any decline in sperm count may be related to male reproductive problems [43]; (3) various semen parameters, including sperm count, have been associated with various environmental factors such as pesticides [44], endocrine disrupting chemicals [45], diet [46], stress [47] and smoking [48]. Therefore, sperm count may reflect the impact of the environment on male health and fertility [49]. Indeed, there is a strict association between lifestyle and male infertility (Figure 1), and available literature has confirmed the potential adverse effects of lifestyle on sperm production. Moreover, several factors induce an additive negative impact on spermatogenesis when exposed in combination with other environmental factors [50].

It is also worthy to note that, among the semen parameters listed above, no spermatogenic endpoint has emerged as a definitive indicator of male reproductive health, and evidence indicates that regulation of male fertility is multifactorial. Therefore, these spermatogenic parameters should be evaluated in conjunction with any available reproductive organ weight, histopathology, and fertility data to best assess male reproductive effects [51].

## 3. Path of NPs into the Reproductive System

Reproductive medicine is a developing field aimed at improving the chances of safe conception and the delivering of healthy babies [52]. Many studies have reported on the use of nanomaterials in detection and targeted therapy related to reproductive cancers [53]. For instance, aptamere-conjugated gold NPs and super-paramagnetic iron oxide NPs are commonly used for the treatment of prostate cancer patients [54,55,56], and also to determine the expression of genes in offspring [56,57,58].

Despite the wide use of NPs in clinical application and reproductive medicine, NPs have the potential to accumulate in tissues and organs, resulting in consequent long-term carcinogenic effects [59]. According to Taylor et al. [60], the accumulation of NPs in somatic cells induces inflammation leading to carcinogenesis, whereas the accumulation of NPs in reproductive cells disturbs the fertility and affects the development of offspring. In drug delivery, penetration of NPs into target cells is important. Use of gold NPs has caused detrimental effects on sperm morphology, motility and DNA of mammalian sperm cells [60,61,62,63].

**Figure 2:**
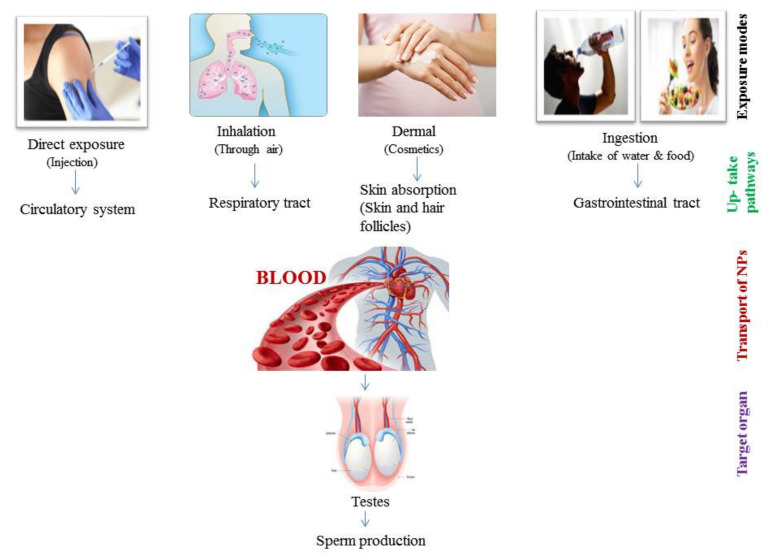
Schematic representation of various routes of nano-particles (NPs) in testes.

Metal and metal oxide NPs are widely used in cosmetology and dermatology. NPs are used for dermatological treatments, for skin care and in diagnostic imaging of skin diseases [64]. Sub-dermal exposure of Ag NPs for 7 and 28 days in male rats showed alterations in sperm number and motility as well as in testosterone (T), luteinizing hormone (LH) and follicle-stimulating hormone (FSH) levels along with histological abnormalities in the testis [65]. In some studies, it is reported that NPs cannot penetrate the skin, while in others penetration of metallic NPs, including iron NPs, is confirmed through hair follicles [66]. This depends on NP size since it has been documented that NPs with 4 nm size can easily penetrate the skin but NPs 45 nm in size can only enter through damaged skin [67]. Rancan et al. [68] also checked the penetration of various sizes of SiO_2_ NPs (291 ± 9 to 42 ± 3 nm) through the skin and found that it was mostly nano-sized particles that showed penetration through damaged stratum cornum. In dermal exposure, NPs can only enter into the epidermis through hair follicles and damaged skin (Figure 2).

Gastrointestinal (GI) exposure of NPs through ingestion is an important absorption pathway. NPs can be absorbed in the GI tract and then enter into the blood, therefore easily reaching the secondary organs and accumulating there (Figure 2) [69]. NPs indirectly enter the body through ingestion as humans take food additives, ingredients and supplements that contain different types of NP [70]. In the food industry, the addition of NPs to various products is increasing, and the most commonly used NPs in food products are ZnO NPs, TiO_2_ NPs, SiO_2_ NPs, and Ag NPs [3]. As a matter of fact, the oral ingestion of different NPs in humans has notably increased in the last decade. Intragastric exposure of TiO_2_ NPs induced immunological dysfunction in mouse testis [71]. Moreover, oral administration of Ag NPs induced reduction in sperm number of male Wistar rats [72,73]. Through ingestion, the daily consumed ranges for various NPs are different. For instance, the daily consumed SiO_2_ NPs is around 126 mg/kg/day for a person with body weight 70 kg [74], though the accepted limit by the European Food Authority for SiO_2_ NPs is 20–50 mg/kg/day for a 60 kg person [75]. Daily ingestion values of Ag NPs and TiO_2_ NPs are around 0.008–0.032 µg/mL and 0.12–12.6 µg/mL, respectively [76]. It is also important to notice that different NPs are characterized by different toxic levels as, for example, TiO_2_ NPs and SiO_2_ NPs are less genotoxic than Ag NPs with the same size range [77].

NPs exposure through inhalation is another important route of exposure through the environment that induces damage to the fetal organs. Inhalation of NPs is linked with molecular alterations in the developmental process and induces deleterious effects on offspring [78]. The ability of NPs to penetrate through the respiratory tract depends upon their size [79,80]. The endocrine activity of the male reproductive system was also disturbed after the exposure to NPs via rich diesel exhaust through inhalation in adult male rats [81]. It was also reported that inhalation of NPs affects the reproductive system of male offspring, including reduced sperm number in F1 males after carbon black NPs exposure [82,83].

Overall, it is known that mononuclear phagocytic cells take up NPs, and in this way NPs enter cells [84]. Absorbance of NPs through dermal, ingestion and inhalation exposure enables nanoparticles to reach the circulatory system and then be translocated to many body tissues and organs, until their accumulation into the reproductive organs (Figure 2) [85], and even into the fetus [86].

Because of their nano size, NPs have the ability to penetrate the biological barriers such as the blood testes barrier (BTB) [34] that provides protection to the reproductive tissues [87]. Therefore, the crossing of NPs induces toxic effects on spermatogenesis [88]. After NP exposure, via different routes, NPs reach the reproductive system, where the main target areas of NPs in males are the epididymis and testis [89]. Sundarraj et al. [90] observed the accumulation of NPs in the testis of mice after iron oxide NP (25 and 50 mg/kg) exposure, documenting the ability of these NPs to cross the BTB and accumulate in the testicular tissue as demonstrated by the presence of iron content. Oral administration of fluorescent europium doped ZnO NPs to mice for 14 days showed their accumulation in testes, indicating the penetration of these NPs through the BTB [91]. Similarly, after intragastric administration of TiO_2_ NPs at the concentration of 2.5, 5 and 10 mg/kg BW in male mice for 90 days, it was observed that NPs crossed the BTB, reached the testes and then accumulated, inducing testicular toxicity resulting in poor quality of sperm, changes in hormone level and testicular lesions [92]. In other studies, contrasting results were reported. For instance, an intramuscular administration of gold core silica shell NPs with 70 nm size in mice showed absence of particles reaching the testes [93]. Similarly, intravenous administration of TiO_2_ NPs at the concentration of 0.1, 1, 2 and 10 mg/kg BW (1 dose/week) for 4 weeks highlighted the accumulation of Ti in the liver but not in the testes of mice [94]. Interestingly, the injection in male mice of Ag NPs with 25 nm size for 4 months via intraperitoneal and intravenous routes, with the aim of investigating the biological fate and potential toxicity of Ag NPs, showed that these NPs were able to cross the BTB and then localize at the testes [95]. Accumulation of Ag NPs was also observed in the basement membrane of testes after oral exposure in male Wistar rats at a dose of 20 µg/kg/day [96]. Zhou et al. [97] also reported the accumulation of Pb Se-NPs in a size dependent manner in male Sprague Dawley rats after intraperitoneal administration at a dose of 10 mg/kg/week that confirmed the transfer of NPs through the BTB.

## 4. Adverse Effects of NPs on the Reproductive System

**Figure 3:**
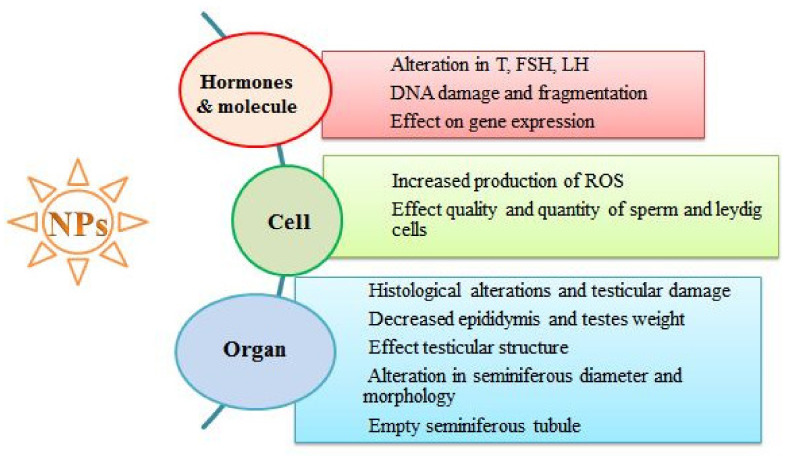
Exposure to NPs and their reproductive toxic effects at various biological levels.

After entering the reproductive system, NPs may induce different deleterious effects at the reproductive organ, cell and hormone levels, as clearly depicted in Figure 3.

### 4.1. Adverse Effects of NPs on Reproductive Organ Weight

After crossing the BTB, NPs reach and accumulate in the reproductive organs, inducing further toxic damage. It is reported that any change in body and organ weight indicates the toxicity induced by chemical exposure [98]. As a matter of fact, the exposure of Ni NPs in healthy adult rats caused reduction in body weight when at various concentrations (5, 15, 45 mg/kg BW), while epididymis to body weight ratio increased in a dose dependent manner [99]. Conversely, no change in body and organ weight was observed after oral exposure to Ag NPs (60 nm) at dose of 15 and 50 µg/kg BW in male Wistar rats [73]. Intravenous administration of Ag NPs (20 nm) at concentrations of 5 and 10 mg/kg BW also induced no alteration in male Wistar rats [100]. A very recent study demonstrated that nanosized Ag NPs caused toxic effects on the reproductive system of male rats. Reduction in body weight was indeed observed after sub-dermal exposure of Ag NPs in male rats when administered at a dose of 50 mg/kg BW for 28 days, whereas decrease in the relative weight of testes and epididymis was found with the same dose exposure for 7 days [65]. Therefore, it is evident that NPs’ impact on body and organ weight is dose and time dependent. Oral administration of ZnO NPs (50, 150, 450 mg/kg) in male mice for 14 days significantly reduced the body weight and increased the relative testicular weight in a dose dependent manner, while the relative epididymis weight was greater at 50 and 450 mg/kg than at a dose of 150 mg/kg ZnO NPs [101]. Hence, different NPs behave differently.

### 4.2. Adverse Effects of NPs on Seminiferous Tubules

The seminiferous tubule is the site for spermatogenesis, and during this process the DNA of spermatogenic cells may be damaged due to ROS production [102]. Exposure to NPs induces histological changes in seminiferous tubules of testicular tissues, leading to testicular injury and reduced sperm production. Intraperitoneal exposure of iron oxide NPs in mice at concentrations of 25 and 50 mg/kg once a week for 4 weeks caused histopathological changes such as sloughing and detachment of germ cells and vacuolization in seminiferous tubules of testicular tissues [90]. Similar intraperitoneal injection of titanium dioxide nanoparticles induced significant increases in the thickness of interstitial spaces, congestion of blood vessels, and detachment of the germinal epithelium from the basement membrane in the seminiferous tubules of adult male albino rats [103]. Indeed, it is well documented that the exposure of NPs, alone as well as in combination with other NPs, induces toxic histological changes in reproductive organs. Distortion in seminiferous tubules and wide spaces among interstitial cells were observed in male rats after exposure to Al_2_O_3_ NPs, while irregularity in the seminiferous tubule shape, empty lumina and reduced thickness of the epithelium lining were observed after exposure to ZnO NPs. Co-administration of Al_2_O_3_ NPs and ZnO NPs induced severe damage both in the seminiferous tubules and basement membrane [104]. The intensity of NP induced toxicity depends also on the dose and time exposure. For instance, exposure to Ag NPs at low and high doses for 7 days induced congestion of blood vessels, detachment of the germinal epithelium and distortion in seminiferous tubules in adult albino rats, but when the exposure duration increased to 28 days a significant reduction in the germinal epithelium and absence of spermatozoa in shrunk seminiferous tubules were observed [105] (Table 2). Dose dependent histological degenerative changes were observed in testicular tissue of adult rats after exposure to Ag NPs at low (2 mg/kg BW) and high (4 mg/kg BW) doses. At low dose, vacuolation in the seminiferous tubule was observed along with reduced number of spermatogenic cell lines, while at high doses of Ag NPs this number was significantly reduced and vacuolation in germinal epithelial cells was particularly noticed, in combination with basement membrane damage and detachment from the surrounding tubules, severe congestion in blood vessels, and few Leydig cells examined in the interstitial tissue [106]. ZnO NP (422 mg/kg/day) exposure for 4 weeks provoked in adult albino rats congestion in blood vessels and detached germinal epithelium from basement membrane, along with absence of spermatozoa in some seminiferous tubules [107]. ZnO NP exposure in albino rats also induced histological abnormalities including disorganization, vacuolation and detachment of germ cells in testicular tissues [88]. Overall, it is ascertained that any damage in the seminiferous tubules may disturb the normal process of spermatogenesis, resulting in the production of abnormal spermatozoa. As supportive references, the table below summarizes literature articles on the exposure of various NPs that induced several histological changes in the seminiferous tubules, provoking consequent reduction in the sperm number by changing the pattern of spermatogenic cells. It is therefore demonstrated that the exposure to different NPs, such as Ni NPs [108], Ag NPs and CeO_2_ NPs [109], induces histopathological changes in the seminiferous tubules leading to decline in sperm number (Table 2).

### 4.3. Adverse Effects of NPs on Sperm Cells

Testicular weight depends on the germ cell mass, and therefore a decrease in testicular weight may be due to the death of germ cells and defects in spermatogenesis [116]. These changes may be due to NPs that, after accumulating in testes, may induce their toxicity by altering sperm morphology, number and viability. The impact of different NPs varies from species to species. The intragastrical administration of TiO_2_ NPs at doses of 10, 50 and 100 mg/kg BW induced reproductive toxicity in male mice. Results revealed that TiO_2_ NPs exposure led to increased sperm malformation and decreased germ cell number [117]. The crossing ability of TiO_2_ NPs through the BTB enables them to reach and accumulate in the testes [92]. Exposure to various NPs caused significant changes in sperm number and induced various abnormalities, as is summarized in Table 3.

All of the literature studies reported in Table 2 and Table 3 revealed that exposure to different NPs induces different toxic effects on quantity, quality and morphology of spermatozoa. Overall, exposure to NPs reduces the sperm count and increases sperm abnormalities. Indeed, sperm morphology is an important indicator against exposure to various occupational and environmental toxicants. Accordingly, it was recently stated that sperm count has been declining for >40 years but the reason is still unknown [142].

### 4.4. Adverse Effects of NPs on Hormones Involved in Sperm Production

Hypothalamic-pituitary-gonadal (HPG) axis is the hormonal system in which the hypothalamus secretes the gonadotropin-releasing hormone that reaches the pituitary gland via blood, inducing the production of LH and FSH, which is further transported to testes. It is known that LH stimulates the Leydig cells to release T in the seminiferous tubules, which are the site of spermatogenesis and where spermatozoa are produced. Moreover, the seminiferous tubules contain an epithelium with a number of scattered cells known as Sertoli cells, which provide support and nutrients to immature sperm cells. NPs can interfere with the levels of secreted hormones by provoking a negative impact on the pituitary and hypothalamus, resulting in the reduction of FSH and LH secretion and a consequent further decline in the T level. Decrease in FSH exacerbates the testicular damage, while the T level reflects the extent of spermatogenic cell depletion as well as the degree of altered spermatogenesis. It was documented that Ni NPs exposure at different doses (5, 15, 45 mg/kg BW) decreased the value of FSH and T, thus indicating the occurrence of testicular injury [99].

Hormones play important roles in regulating the development of the reproductive system as well as in controlling its activities. In the last two decades, several research works have focused on the endocrine disrupting chemicals (EDCs) regarding reproductive health [113]. Some hormones may be altered after exposure to different NPs. It was reported that intravenous administration of Ag NPs at low dose (1 mg/kg/dose) in male CDI mouse serum significantly increased testosterone level [126]. Testosterone level was also highly increased in male rats when gold nanoparticles (Au NPs) were intraperitoneally administered at concentrations of 25, 50, and 100 ppm. Besides the augmented T level, highly significant increases in LH and FSH levels were also observed in male rats after 10 days of Au NPs exposure, in combination with increased infertility [143]. Conversely, it was observed that exposure to some NPs reduced the T level, as found in mice challenged with TiO_2_ NPs (300 mg/kg), with further evidence of the beneficial effects induced by quercetin [144]. However, it is worthy of note that during prepubertal development of male Wistar rats the levels of FSH, LH and T did not change after daily exposure to Ag NPs at doses of 15 and 30 µg/kg [130]. It was also observed that exposure to Ag NPs at doses of 0, 10 and 50 mg/kg BW for the duration of 7 and 28 days in male rats induced dose and time dependent changes in LH, FSH and T level [109]. Similarly, oral administration of Al_2_O_3_ NPs and ZnO NPs at doses of 70 and 100 mg/kg BW/day for 75 days in Wistar male albino rats induced, respectively, reduction in TSH and T levels, and increases in LH and FSH levels [104]. Therefore, it may be stated that changes in hormone levels might be influenced by various factors including size, type and exposure time of NPs. Overall, decrease in T level leads towards testicular injury, while the rise in LH and FSH level might be related to the onset of negative feedback mechanisms [113].

## 5. Conclusions

This review recognizes that some nanoparticles (NPs) may act as reproductive toxicants depending on several factors (i.e., type of NPs, exposure route and duration), and induce damage to the male reproductive system by affecting the seminiferous tubules and spermatogenesis [145,146]. This is mainly due to the fact that NPs can easily enter the blood circulatory system and reach the testes by crossing the blood testes barrier. The bioaccumulation of NPs in the testes causes seminiferous tubule histopathology and severely affects the sperm number, motility and morphology. Moreover, NPs also induce disturbances to the Leydig cells, causing decline in the testosterone level with consequent testicular injury and reduced sperm production. Therefore, more investigation is needed to better elucidate the safety issue of different NPs on reproductive health. Tighter national and international regulations should also be approved for the use of nanotechnology products.

## Figures and Tables

**Table 1:** Normal semen parameters with values at 95% confidence intervals (CI), according to the WHO.

Parameters	Values	95% CI
Volume	1.5 mL	1.4–1.7
Sperm concentration	15 million spermatozoa/mL	12–16
Total number of sperm per ejaculation	39 million spermatozoa	33–46
Sperm Morphology	4% normal forms	3–4
Sperm vitality	58% live	55–63
Progressive motility	32%	31–34
Total (progressive and nonprogressive sperm motility)	40%	38–42

**Table 2:** NPs induce histological abnormalities in sperm and seminiferous tubules, disturbing sperm production.

Test Material	Histological Evaluation		References
Nanoparticles	Sperm Morphology	Seminiferous Tubules	
Nickel nanoparticles		Increased number of abnormal sperms in epididymis, cell apoptosis, no proper arrangement of germinal cells, large gap in lumen of seminiferous tubules	[108]
Silver nanoparticles	Different sperm cell abnormalities including double head, long tail, No hook or wrong hook attachment	Adverse hypertrophic seminiferous tubules	[110]
	Development of abnormal spermatids	Atrophy in seminiferous tubules, necrosis and degradation of spermatogenic cells, and in spermatogonia and Sertoli cells, ultra-structural alterations	[96]
		Shrunken seminiferous tubules, loss of sperms in seminiferous tubules, presence of multinucleated giant cells	[105]
	Sperm with coiled, bent and headless tail, detached head	Increased desquamation in the lumen	[111]
Zinc oxide nanoparticles		Detachment (D), sloughing (S), vacuoles (V) in seminiferous tubules, loss of spermatids, disorganization of germ cells, vacuolization in germinal epithelium	[112]
Titanium dioxide nanoparticles	Amorphous head, double tails of sperm, double head with fused tails, short and knobbed hook	Depletion and necrosis in spermatogenic cells, vacuolation	[113]
Zinc oxide nanoparticles and titanium dioxide nanoparticles	Massive head, double hook, double tail with pin head, folded spermatozoa	Seminiferous tubules with variation in size, depletion in spermatogenic cells, necrosis in spermatogenic cells, increased luminal width, congestion in interstitial blood vessels	[28]
Cerium oxide nanoparticles		Necrosis in seminiferous tubules, apoptosis in interstitial tissues, loss of spermatozoa, decline in the number of Sertoli cells, Leydig cells, and spermatids	[109]
Silica-gold nanoparticles		Empty seminiferous tubules	[93]
Aluminum oxide nanoparticle		Vacuolization, edema in interstitial cells and congestion in blood vessels, necrosis in spermatogenic cells	[114]
Anatase titanium dioxide nanoparticles	Coil and folded sperm with missing cap		[115]

**Table 3:** Exposure to NPs and effect on spermatogenesis.

Test Material	Experimental Model	Age	Exposure of NPs				Findings	Reference
			Size	Route	Doses	Days		
Carbon black nanoparticles	ICR male mice		14, 56, 95 nm	Intratracheally	0.1 mg	10 times/week	T level increase (14, 56 nm) DSP decreased	[118]
Titanium dioxide nanoparticles	Male Wistar rats		21 nm	Intravenously			TSH decreased, Sperm count reduced	[119]
	Adult male albino rats	180–200 g		Orally	100 mg/kg/day	8 weeks	Increased sperm abnormalities, motility, curved sperm tail, decreased sperm viability, sperm count and T level	[120]
	C57BL/6Jgpt delta mice	8 weeks		Intravenously	0, 2, 10 mg/kg/BW/week	4 weeks Kill 9 days after last injection	Number of sperm head reduced in testes and cauda epididymis, toxic effect on sperm quality	[121]
	Albino male mice	4 months	10 nm	Intraperitoneally	5, 10, 50, 100, 150 mg/kg/BW/day	14 days		[122]
	Adult male Wistar rats	2 months 150–250 g	18 nm	Intraperitoneally	1 mL of 30, 50 mg/kg/dos	Alternate days for 21 days	Decreased T level, increased LH, no change in FSH	[123]
	NMRI mice	6–8 weeks 25–30 g			300 mg/kg	35 days	T level decreased, sperm motility and number changed	[124]
	Adult Swiss male mice	11–15 weeks 28–32 g	<25 nm	Intraperitoneally	TiO_2_NPs: 9.38, 18.75, 37.5, 75 mg/kg b.w	Daily for 35 days	Changes in sperm motility, number, increased abnormalities, reduced LH level	[28]
Titanium nanoparticles	MaleC57BL/6J	8 weeks		Intravenously	10, 50 mg/kg (single injection)	1 3 9 days	Decreased sperm motility, sperm number not reduced in cauda epididymis and testes, blockage of blood vessel at higher dose (50 mg) After 3 days oral administration: Sperm motility reduced, no change in spermatozoa in cauda epididymis	[125]
				Orally	20, 100 mg/kg (single dose)			
Silver nanoparticles	Male Wistar rats	Postnatal day PND 23	60 nm	Orally	15, 50 µg/kg BW	Once/day PND 53 PND 90	PND 90: Reduction in sperm count and TSH PND 53 and 90: sperm reserve in epididymis	[73]
	Male Wistar rats	200–250 g 45–50 days	70 nm	Orally	25, 50, 100, 200 mg/kg/day	Every 12 h in 48 days	Decrease in number of spermatogenic cells at 200 mg/kg dose, primary spermatocytes, spermatids and spermatozoa reduced at dose of 50, 100, 200 mg/kg	[72]
	Male Wistar rats	14 weeks	20 nm, 200 nm	intravenously	5, 10 mg/kg body mass (20 nm) or 5 mg/kg (200 nm)	24 h, 7 days, 28 days	Reduction in sperm count, increased abnormal spermatozoa after 1–4 weeks than 24 h	[100]
	Male ICR mice	8 days old	15 nm	Sub-cutaneously	1, 5 mg/kg/dose (5 doses for every 3 days)	PND 28, PND 42, PND 63, PND 100	On PND 42 and 63: Sperm abnormalities increased at 5 mg On PND 100: Epididymal level of sperm decreased at 5 mg	[126]
	Male rats	100–150 g	100 nm	Sub-dermally	0, 10, 50 mg/kg BW	7 days, 28 days	Dose and time dependent changes in T, LH and FSH Decrease in sperm velocity parameters	[65]
	Male Wistar rats		70 nm	Orally	25, 50, 100, 200 mg/kg/day	45 days	Reduction in number of Leydig cells, T level, FSH, sperm motility, number of immotile sperms increased in a dose dependent manner	[127]
	Male CD1 mice	4–5 weeks	10 nm	Intravenously	1 mg/kg 5 times (once every 3 days)	15, 60 and 120 days	No change in sperm count, motility and fertility indices, T level increased on day 15	[128]
	Male Swiss Webster mice	30–35 g	56.67 ± 9.77 nm	Intraperitoneally	20, 41, 82 mg/kg	After 24 h from injection	Decreased concentration of sperm, increased number of abnormal sperms at all doses	[129]
	Male Wistar rats	10–12 weeks	5-20 nm	Orally	20 µg/kg/day	90 days	No changes	[96]
	Male Wistar rats		60 nm	Orally	15, 30 µg/kg/day	PND 23, PND 58	At both doses: Sperm abnormalities increased, no changes in T, LH and FSH	[130]
	Male adult Wistar rats	200–250 g	60–80 nm	Intraperitoneally	30, 125, 300 mg/kg	Single dose	Sperm count and vitality reduced, decreased spermatogonia, Leydig and Sertoli cells	[131]
Polyvinil pyrrolidone coated silver nanoparticles	Adult male Sprague Dawley rats	240–280 g	20–30 nm	Orally	50, 100, 200 mg/kg/day	90 days	No significant changes on sperm motility At 100 mg: Number of abnormal sperms increased	[111]
Zinc oxide nanoparticles	Adult albino mice	3 weeks	80 nm	Orally	0, 150, 350 mg/kg/BW	15 days		[132]
	Adult male NMRI mice			Orally	0, 5, 50, 300 mg/kg/day	35 days	At 50 and 300 mg: Significant alterations in sperm number, sperm motility and abnormality of epididymal sperms	[133]
	Adult male Wistar rats	6–8 weeks 180–220 g	10–30 nm	Intraperitoneally	0, 50, 100, 150, 200 mg/kg/BW/day	10 days	More than 50 mg: Viability and number of sperms reduced More than 100: Quality of sperms decreased	[134]
	Mature NMRI mice	28–32 g	20 nm	Intraperitoneally	0, 250, 500, 700 mg/kg/day	1 week after single injection	No change	[135]
	Wistar Male albino rats	4–5 months 160–170 g	100 nm	Orally	100 mg/kgBW/day	75 days	Number of sperms and motility decreased, reduced T while LH and FSH increased	[104]
	Adult Swiss male mice	11–15 weeks 28–32 g	<100 nm	Intraperitoneally	9.38, 18.75, 37.5, 75 mg/kg b.w/day	35 days	TSH level increased	[28]
Manganese dioxide nanoparticles	Male Wistar rats	180 ± 5 g 8–10 weeks	<5 mm 25–85 nm	Sub-cutaenously	100 mg/kg	Once a week for 4 weeks	At both doses: Increased number of immotile sperms, decreased number of epididymal sperm, spermatogonia and spermatocytes, no change in T and FSH	[136]
Graphene Oxide nanoparticles	Adult Wistar rats	10–12 weeks	0.8–2 nm	Intraperitonealy	0.4, 2 and 10 mg/kg BW	Repeated exposure for 15 and 30 days	Reduced epididymal sperm count, motility, increased sperm abnormality	[137]
Nickel nanoparticle	Sprague Dawley rats	80–100 g	90 nm	Orally	0, 5, 15, 45 mg/kg	10 weeks	Decreased FSH, T but LH increased, sperm motility reduced	[99]
Lead sulphide nanoparticles	Sprague Dawley rats	180–240 g) 12–14 weeks	30 nm	Orally	0, 25, 50, 100 mg/kg	5 days/week for 6 weeks	Decreased sperm count and rate of sperm survival	[138]
Lead selenide nanoparticles	Male specific pathogen free Sprague Dawley rats	170–200 g 6–7 weeks	8 nm, 30 nm, 70 nm	Intraperitoneally	10 mg/kg/week	60 days	At 8 nm and 30 nm: Increased sperm abnormalities, poor quality and quantity of sperms, T and FSH more reduced in 8 nm as compared to 30 nm and 70 nm in a size dependent manner	[97]
Gold nanoparticles	Male bulb –c mice	5 weeks	10–30 nm	Intraperitoneally	40 and 200 µg/kg/day	7 and 35 days	Increased number of abnormal spermatozoa, reduced sperm motility and altered sperm morphology	[139]
Aluminium oxide nanoparticles	Wistar Male albino rats	4–5 months 160–170 g	50 nm,	Orally	70 mg/kgBW/day	75 days	Decreased semen characteristics: number of sperms and its motility, TSH, T, increased FSH, LH	[104]
Cerium oxide nanoparticles	Male bulb –c mice	8 weeks 30–35 g	Less than 10 nm	Intraperitoneally	100, 200 and 300 μg/kg body weight	Three times/week for 5 weeks	Decreased T, FSH and LH, reduced sperm count and motility, increased sperm abnormality	[140]
Titanium dioxide nanoparticles and α-quartz particles	Male mice C57BL/6J	9 weeks		Intratracheally	63 µg	Once weekly for seven weeks	No effect on sperm count and T level	[141]

Testosterone (T); Daily Sperm Production (DSP); Thyroid Stimulating Hormone (TSH); Luteinizing Hormone (LH); Follicle Stimulating Hormone (FSH); Postnatal Day (PND).
